# Calcium Homeostasis: A Potential Vicious Cycle of Bone Metastasis in Breast Cancers

**DOI:** 10.3389/fonc.2020.00293

**Published:** 2020-03-10

**Authors:** Zhengfeng Yang, Zhiying Yue, Xinrun Ma, Zhenyao Xu

**Affiliations:** ^1^Shanghai Institute of Immunology Center for Microbiota & Immune Related Diseases, Institute of Translational Medicine, Shanghai General Hospital, Shanghai Jiao Tong University School of Medicine, Shanghai, China; ^2^Department of Urology, Renji Hospital, School of Medicine, Shanghai Jiao Tong University, Shanghai, China

**Keywords:** calcium homeostasis, calcium channels, vicious cycle, tumor progression, osteoclast activation, bone metastasis

## Abstract

Cancers have been considered as one of the most severe health problems in the world. Efforts to elucidate the cancer progression reveal the importance of bone metastasis for tumor malignancy, one of the leading causes for high mortality rate. Multiple cancers develop bone metastasis, from which breast cancers exhibit the highest rate and have been well-recognized. Numerous cells and environmental factors have been believed to synergistically facilitate bone metastasis in breast cancers, from which breast cancer cells, osteoclasts, osteoblasts, and their produced cytokines have been well-recognized to form a vicious cycle that aggravates tumor malignancy. Except the cytokines or chemokines, calcium ions are another element largely released from bones during bone metastasis that leads to hypercalcemia, however, have not been well-characterized yet in modulation of bone metastasis. Calcium ions act as a type of unique second messenger that exhibits omnipotent functions in numerous cells, including tumor cells, osteoclasts, and osteoblasts. Calcium ions cannot be produced in the cells and are dynamically fluxed among extracellular calcium pools, intracellular calcium storages and cytosolic calcium signals, namely calcium homeostasis, raising a possibility that calcium ions released from bone during bone metastasis would further enhance bone metastasis and aggravate tumor progression via the vicious cycle due to abnormal calcium homeostasis in breast cancer cells, osteoclasts and osteoblasts. TRPs, VGCCs, SOCE, and P2Xs are four major calcium channels/routes mediating extracellular calcium entry and affect calcium homeostasis. Here we will summarize the overall functions of these four calcium channels in breast cancer cells, osteoclasts and osteoblasts, providing evidence of calcium homeostasis as a vicious cycle in modulation of bone metastasis in breast cancers.

## Introduction

Cancers have been considered as a worldwide health problem for years. In 2018, 18 million new cases of cancers were diagnosed and around 9.6 million death was reported, which accounts for the second leading cause of death in the world (1). Cancer metastasis has been well-recognized as one of the major causes for cancer progression as well as the high cancer mortality rate, especially the bone metastasis (2). Multiple type of cancers develop bone metastasis, including breast, prostate, thyroid, lung, renal, melanoma, head and neck, gastrointestinal tract and ovarian, from which breast cancer and prostate cancer are two typical types that exhibit highest percentage of bone metastasis rate, with ~70% in both breast cancers and prostate cancers (3).

Bone metastasis is a process by which primary tumor cells spread to the bone through the bloodstream or lymph vessel. The migrated/metastasized tumor cells then proliferate in the bone and enhance abnormal osteoclastogenesis or osteoblastogenesis (3). In breast cancers, bone metastasis promotes osteoclast activation and leads to over-activated osteolysis. The osteolytic lesions provide comfortable niches that multiple cells including tumor cells, osteoblasts, and osteoclasts communicating with each other, resulting in continuous tumor growth in the bone as well as in the primary sites that forms a vicious cycle (4). The vicious cycle is frequently observed in the late-stage of breast cancers (stage IV) and is a multi-step processes require numerous types of cells to participate in. Briefly, breast cancer cells in the primary sites invade in the surrounded blood vessels probably via the epithelial–mesenchymal transition (5). The infiltrated tumor cells survive in the vessel with interacting with host cells, including red blood cells, neutrophils, platelets, etc., and migrate to different sites for organ invasion. Bone metastasis is occurred when the tumor cells in the vessel migrate to the bone, where the tumor cells undergo mesenchymal-epithelial transition (6, 7). Also, neovascularization is accompanied with bone metastasis, which the endothelial cells could be activated by angiogenic factors secreted by tumor cells and bone marrow (8, 9). This vasculogenesis has also been believed to facilitate bone metastasis of breast cancer cells (10). Other cells, include adipocytes, myeloid cells and Treg, all have been shown to promote bone metastasis via direct cell-cell contacts or indirect secretion of cytokines.

**Figure 1:**
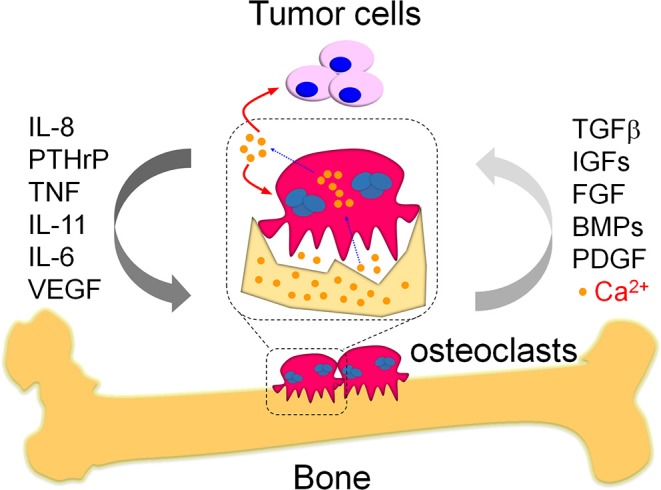
The vicious cycle in bone metastasis. Bone metastasis is highly occurred in breast cancer patients, which results in vicious cycles to further deteriorate the primary tumor burden and promote tumor growth in bone. One kind of the well-known vicious cycles is a systemic response in tumor cells and osteoclasts. Briefly, the migrated tumor cells produce multiple cytokines and eventually enhance osteoclast differentiation as indicated. The pathologically activated osteoclasts heavily destroy bone that release numerous growth factors from damaged bone matrix, which would in turn promote tumor cell growth and therefore form the vicious cycle between tumor cells and osteoclasts during bone metastasis. Except growth factors, the damaged bone also releases large amounts of calcium ions. These calcium ions were specifically released in the sealing zones where mature osteoclasts tightly attached with the bone. The mature osteoclasts also exhibit specialized plasma membrane in the sealing zone, named as ruffled border. The ruffled border facilitate the release of calcium ions from bone via the vesicular transport in osteoclasts. The released calcium ions might further aggravate bone metastasis via modulation of the activity in tumor cells and osteoclasts, which is a potential vicious cycle required further efforts to elucidate.

Numerous chemokines or cytokines facilitate bone metastasis through either the autocrine or the paracrine pattern, which have been well-summarized in these two decades (4, 11–13). Recently, calcium ions have also been reported to greatly modulate cancer progression (14, 15), however, have not been well-summarized yet. Calcium ions are one of the important second messengers that decode extracellular stimulation and thus regulate biological functions (15, 16). Unlike other second messengers, calcium ions are not produced by cell itself, but all come from extracellular calcium entry, which forms calcium homeostasis in a cell (17). Calcium homeostasis is largely affected during bone metastasis as bone is the major organ for calcium storage. Briefly, the abnormally enhanced osteoclastogenesis in cancer patients would increase bone resorption and lead to huge amounts of calcium release into blood as 99% calcium is stored in the bone (4, 18). The released calcium would then further affect bone metastasis via the abnormal calcium homeostasis in tumor cells, osteoclasts or osteoblasts, forming another potential vicious cycle for bone metastasis (Figure 1). Indeed, cancer patients have been observed to suffer hypercalcemia (19). Importantly, the survival rate of cancer patients with hypercalcemia is largely reduced (19), specifically the 1-year survival rate is below 30%, indicating the importance of the blood calcium levels in modulating cancer progression. These clinical observations raise an important question that how hypercalcemia worsens the progression of tumors, by targeting tumor cells, osteoclasts, osteoblasts, or the communication niches? Here we will take the breast cancer as the typical representative to specifically overview recent efforts on understanding the functions and mechanism of calcium homeostasis in breast cancer cells, osteoclasts, osteoblasts and thus bone metastasis.

## Calcium Homeostasis in the Cells

Calcium ions are well-characterized as the second messenger for multiple signaling pathways that modulate numerous biological functions, including muscle contraction, apoptosis, neural transmission, cell differentiation, and cell metabolism, etc. (16, 20), which is largely dependent on its hundreds of patterns, such as calcium release, calcium oscillations, calcium spikes, etc. These patterns are the result of calcium fluxes among cytosolic calcium ions, intracellular calcium storages and extracellular calcium pools. Normally, the extracellular calcium pool is maintained at the concentration of 1–2 mM, and the calcium level in the intracellular calcium storage, specifically the endoplasmic reticulum (ER), is around 100–400 μM. Whereas, the cytosolic calcium level is about 100 nM (17). Such gradient makes the calcium signaling be dynamically modulated without hurting the cell viability that would be affected by abnormal calcium levels in the cytoplasm. Indeed, the half-life of IP3, the major messenger inducing calcium signals, is 60 s (21), a pretty short period sufficiently activating calcium release from ER, transducing downstream signals, but not abnormally increasing the cytosolic calcium level that would be toxic to the cells.

**Figure 2:**
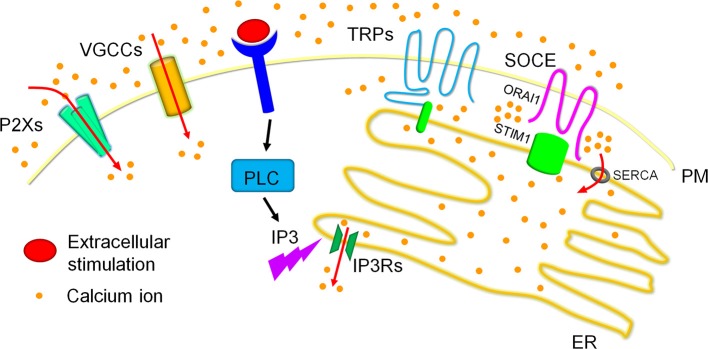
Calcium homeostasis in cells. Calcium ions are dynamically fluxed among extracellular calcium pools, intracellular calcium storages and the cytoplasmic matrix. Four major calcium channels/routes mostly on the plasma membrane (PM) mediate extracellular calcium entry, including P2Xs, VGCCs, TRPs, and SOCE, of which TRPs and SOCE have been reported to modulate dynamic calcium fluxes in response to the reduction of calcium concentrations in the ER calcium storage. Particularly, extracellular stimulation activated PLC family members result in the production of IP3. The produced IP3 swiftly binds to IP3Rs on the ER membrane and triggers ER calcium release, which leads to the reduction of ER calcium storage. The reduced ER calcium storage activates calcium release-activated channels (CRAC) for extracellular calcium entry to refill the ER calcium ions and therefore sustain cytosolic calcium signals. TRPs especially TRPCs and SOCE have been reported to mediate CRAC. TRPCs have been shown to associate with STIM1 on the ER membrane to generate the channel for calcium entry. SOCE, the best recognized route for CRAC in recent decade, is mediated by ORAI1 on the PM and STIM1 on the ER membrane. Briefly, STIM1 is inactivated when calcium concentrations are maintained highly in the ER calcium storage, and underwent conformational change after the reduction of ER calcium contents, specifically initiated after ~25% reduction. The conformational changed STIM1 are then oligomerized and redistributed to the ER-PM contact to interact with the clustered ORAI1, forming the calcium channel for extracellular calcium entry. These entered calcium ions can enter the ER calcium storage via the SERCA on the ER membrane.

Particularly, calcium fluxes could be initiated by extracellular stimulators, such as G-protein coupled receptors and their ligands. The activated signals were then transduced to promote IP3 production via PLC family members. IP3 can bind to its receptors IP3Rs on the ER and therefore induces calcium release from ER (22) (Figure 2). These released calcium activates downstream signals by binding to the targeting proteins, like Ca^2+^ /calmodulin-dependent protein kinase (CAMK) and calcineurin, and eventually induces the nuclear translocation as well as the transcriptional activity of NFAT family members (23). Importantly, the calcium-NFAT axis is specifically modulated in particular cell types (24, 25), like T cells and osteoclasts, where calcium release is continuously occurred and maintained with a high frequency but a low amplitude, namely calcium oscillations, a more efficient way to facilitate NFAT activation.

Calcium oscillations are one of the typical representatives of calcium homeostasis in a cell. For instance, in osteoclasts the RANKL/RANK signaling recruits TRAF6 and activates PLCγ2 (26, 27), after which the phosphatidylinositol biphosphate (PIP2) is converted into IP3 and DAG. The produced IP3 then bind to IP3Rs as mentioned above, triggering calcium release from ER that initiates the calcium signal but also reduces the ER calcium storage. To sustain the calcium oscillations, the reduced calcium storage then promotes the activation of STIM1 located in the ER membrane that induces extracellular calcium entry by association with ORAI1 on the plasma membrane, which refills the reduced or depleted ER calcium storage and facilitates further ER calcium release (28–31) (Figure 2). The continued cycles of these calcium fluxes form calcium oscillations and sustain the NFAT activation (24).

Though STIM1/ORAI1 mediated calcium entry is one of the most important routes for extracellular calcium entry in non-excitable cells reported in a decade, multiple calcium channels are responsible for extracellular calcium entry, mainly including four major families. The dynamic calcium levels modulated by these channels have been reported to be critical signals for cancer cells viability and tumor formation (Table 1). In the following parts, we will summarize how these calcium channels modulate tumor progression and their potency in regulation of bone metastasis in breast cancers.

**Table 1:** Mammalian calcium channels and their functions in breast cancer cells and osteoclasts.

**Family**	**Members**	**Functions in breast cancer cells**	**Functions in osteoclasts**	**References**
TRPC	TRPC1	Proliferation ↑ TGFβ-induced EMT↑	I-mfa deficient osteoclast differentiation↑	(32–36)
	TRPC2	/	/	/
	TRPC3	/	/	/
	TRPC4	/	/	/
	TRPC5	Drug resistance ↑ Angiogenesis ↑	/	(37–39)
	TRPC6	Proliferation, survival and migration ↑	/	(40)
	TRPC7	/	/	/
TRPV	TRPV1	Drug resistance ↓ metastatic bone pain ↑	Osteoclast differentiation and activation ↑	(41–44) (45, 46)
	TRPV2	/	Calcium oscillations and osteoclastogenesis ↑	(47, 48)
	TRPV3	/	/	/
	TRPV4	Apoptosis and oncosis ↑ Actin reorganization and tumor invasion ↑	Late-stage osteoclast activation ↑	(49–51) (52–54)
	TRPV5	/	Size and number ↓ Bone resorption ↑	(55, 56)
	TRPV6	Proliferation ↑ Drug resistance ↓	Size and number ↓ Bone resorption ↓	(57–59) (60)
TRPM	TRPM1	/	/	/
	TRPM2	Cell viability ↑	/	(61)
	TRPM3	/	/	/
	TRPM4	/	/	/
	TRPM5	/	/	/
	TRPM6	/	/	/
	TRPM7	Metastasis ↑ Mesenchymal feature ↑ Proliferation ↑	/	(62–65)
	TRPM8	EMT and migration ↑	/	(66)
TRPA	TRPA1	Apoptosis ↓ Drug resistance ↑	/	(67)
TRPP	TRPP2	Drug resistance ↑	/	(68)
	TRPP3	/	/	/
	TRPP5	/	/	/
TRPML	TRPML1	Tumor growth and migration ↑	Lysosomal functions and osteoclast activation ↑	(69, 70)
	TRPML2	/	/	/
	TRPML3	/	/	/
VGCC	Cav1.1	/	/	/
	Cav1.2	/	/	/
	Cav1.3	Proliferation ↑	/	(71)
	Cav1.4	/	/	/
	Cav2.1	/	/	/
	Cav2.2	/	/	/
	Cav2.3	/	/	/
	Cav3.1	Proliferation ↓ Apoptosis ↑	/	(72)
	Cav3.2	Proliferation ↑	/	(73)
	Cav3.3	Proliferation ↑	/	(74)
SOCE	STIM1	Migration and metastasis ↑ EMT↑	Calcium oscillations ↑	(75–80)
	ORAI1	Focal adhesion, migration and invasion ↑ Bone metastasis ↑	Fusion and differentiation ↑	(76, 81–84)
P2X	P2X1	/	/	/
	P2X2	/	/	/
	P2X3	/	/	/
	P2X4	/	/	/
	P2X5	/	/	/
	P2X6	/	/	/
	P2X7	Proliferation ↑ Apoptosis ↓ Migration, metastasis ↑	Fusion and differentiation in pathological conditions ↑	(85–89) (90–95)

*↑, indicates the functions have been upregulated; ↓, indicates the functions have been downregulated*.

## Transient Receptor Potential (TRP) Channels

The TRP channels are six transmembrane channels located mostly on the plasma membrane and sense multiple physiological stimuli, including vision, taste, olfaction, hearing, touch, thermo- and osmo-sensation (96). In response to these stimuli, the TRPs act as cation channels for multiple ions, from which calcium is one of the best recognized ions (97). The mammalian TRPs are a large superfamily that contains six subfamilies and around 30 members, including TRPCs, TPRVs, TRPMs, TRPA1, TRPPs, and TRPMLs (96, 98), which leads to a diverse cation selectivity in multiple cells, from neuron to non-neuron cells. Though TRPs exhibit multiple activation patterns, they share one common mechanism coupled to phospholipase C (PLC) activation and are responsible for extracellular calcium entry. For instance, activation of PLC by upstream signals like G-protein couple receptors would lead to calcium release from ER, the reduced calcium in the ER would then activate TRPs for extracellular calcium influx, similar as the store-operated calcium entry, to sustain further calcium signals in the cells (97). The exact mechanism linked PLC and TRPs activation is still not fully clarified and required to be discussed case by case, which has been well-described elsewhere (96, 99). Following we will summarize recent advances in understanding the function of TRPs in regulation of tumor progression and bone metastasis.

### TRPCs

#### TRPCs in Breast Cancers

The mammalian TRPCs contain seven members, from TRPC1 to TRPC7, from which human TRPCs contain six members as human TRPC2 is a pseudogene. TRPCs have been believed to promote tumor cell proliferation and survival in multiple tumor cells, including colon cancers, non-small cell lung cancers, glioma, gastric and esophageal cancers (100). Importantly, TRPCs have been shown to have broad functions during breast tumor progression. TRPCs have been shown to express in multiple solid tumors. In breast cancers, TRPC1 and TRPC6 are found to be highly expressed in human breast ductal adenocarcinoma compared to the adjacent non-tumor tissues (32, 101, 102), indicating the potential roles of these two TRPCs in modulation of breast tumor progression. Several studies found that TRPC1 promotes breast cancer cell proliferation and facilitates TGFβ-induced epithelial-mesenchymal transition (EMT) (32–35), suggesting that TRPC1 is an essential signal for breast tumor growth and metastasis. Indeed, TRPC1 is expressed highly in basal breast cancer cell lines and tumor tissues from patients suffering basal breast cancers, especially those accompanied with lymph node metastasis. Mechanistically, TRPC1 is required for AKT activation to induce HIF1α expression, and thus promotes EMT. Similarly, TRPC6 is also highly expressed in breast cancer cell lines compared to normal control. Silencing TRPC6 largely reduces proliferation, survival and migration in breast cancer cell lines (40), which might be due to reduced expression of ORAI1 and ORAI3 in TRPC6 deficient cells. TRPC5 is another TRPC that has been well-addressed in breast cancers progression. Unlike TRPC1 and TRPC6, TRPC5 has been identified to mediate chemotherapeutic resistance in breast cancers. When breast cancer cells or patients are treated with adriamycin, TRPC5 is upregulated in extracellular vesicles, which is believed to be responsible for the drug resistance (37, 38). Further studies show that TRPC5 also mediates autophagy by the CaMKKβ/AMPKα/mTOR pathway and therefore enhances the adriamycin resistance in breast cancers (39). In addition, TRPC5 is also upregulated in breast cancers and mediates angiogenesis during tumor progression, which is another important aspect that TRPC5 promotes breast cancers metastasis. So far, no more other TRPCs have been reported to affect breast tumor formation and development. TRPCs could be classified as four subsets according to their amino acids similarity, including TRPC1, TRPC2, TRPC3/6/7, and TRPC4/5 (96). Considering that TRPC1, TRPC5, and TRPC6, representatives of the different three subsets, all mediate calcium influx and are required for tumor cells proliferation and metastasis in breast cancer cells, the other TRPCs, specifically TRPC3, TRPC4, and TRPC7, might be also important modulators of breast cancer progression in certain scenarios. Further studies are required to elucidate the expression profiles of these TRPCs in different stages of breast cancers, which might give evidence to elucidate how these TRPCs independently or synergistically modulate breast cancers progression.

#### TRPCs in Osteoclasts

Till now little has been known about TRPCs in regulation of osteoclastogenesis except TRPC1 (36). TRPC1 knockout mice exhibit normal osteoclastogenesis and bone mass in physiological conditions. However, deficiency of I-mfa, the inhibitor of Trpc1, increases osteoclast differentiation and reduces bone mass. Importantly, I-mfa and Trpc1 double knockout mice exhibit largely restored osteoclastogenesis and bone mass, suggesting the activation of Trpc1 is required for normal osteoclast differentiation and the maintenance of bone density. These observations also indicate that Trpc1 channel in mice is inactivated in physiological conditions. Whether Trpc1 affects osteoclast development and functions in pathological conditions like tumors is required to be elucidated. Moreover, one might also need to consider the compensation effects among TRPCs due to their functional similarity when understanding a specific TRPC channel in modulation of osteoclastogenesis.

Taken together, TRPCs, especially TRPC1, play essential roles in modulation of tumor progression and osteoclast activation. Considering TRPC1 in osteoclasts is inactivated in physiological conditions and required for EMT in breast cancers, it will be interesting to examine whether TRPC1 is activated when bone metastasis is occurred in breast cancers. Moreover, such activation probably will lead to enhanced bone metastasis as TRPC1 promotes calcium influx that benefits both cancer cells metastasis and osteoclasts activation.

### TRPVs

#### TRPVs in Breast Cancers

The mammalian TRPVs contain six members, named as TRPV1 to TRPV6. Unlike TRPCs, most TRPVs reported seem to act as the tumor suppressor in breast cancers. So far, TRPV1, TRPV4 and TPRV6 have been well-studied in breast cancers progression. It has been reported that TRPV1 activation via capsaicin together with MRS1477 largely reduces MCF-7 viability (41), which is not observed in primary breast epithelial cells, indicating TRPV1 is a potential drug target for treating breast cancers without affecting normal cells. Moreover, activation of TRPV1 also increases the anti-tumor efficiency of clinical drugs like doxorubicin probably via aggravating the ROS induced apoptosis (42). Importantly, TRPV1 is expressed in neurons and senses signals of pain (103), whereas tumor-induced bone pain is a severe clinical condition that needs to be addressed (104). Tong et al. show that TRPV1 is activated by formaldehyde secreted by the cancer tissues and induces metastatic bone cancer pain, especially in the condition of tumor acidic microenvironment (43, 44). These observations raise a complicated network among tumor cells, neurons, bones and the extracellular calcium pool in the metastatic bone microenvironment, which is required efforts to be further studied.

The role of TRPV4 in breast cancers is much complicated, making it difficult to be a potential drug target at present. Particularly, activation of TRPV4 induces both cell death and metastasis in breast cancer cells. One study reported that pharmacological activation of TRPV4 by GSK1016790A drastically enhances tumor cells death mainly via two routes: apoptosis mediated by PARP-1 cleavage and oncosis accompanied with a rapid decrease of intracellular ATP production (49). Interestingly, the expression of TRPV4 is correlated with poor clinical outcomes in breast cancers (50). Further studies show that the expression of TRPV4 in breast cancer cells lead to the actin reorganization and therefore promotes breast cancer cell softness and tumor invasion without affecting cell proliferation (50, 51). Similarly, TRPV4 is also required for actin remodeling in endothelial cells and thus promotes angiogenesis and tumor progression (51). All in all, more efforts are required to precisely understand the molecular mechanism of TRPV4 in either enhancing tumor cell death or cytoskeleton reorganization, which might provide potential strategies for treating breast cancers by targeting TRPV4 in the future.

Unlike TRPV1 and TRPV4, TRPV6 has been shown to positively promote breast cancers progression. TRPV6 is widely upregulated in multiple malignant cells including breast cancer cells (57). Specifically, TRPV6 is highly expressed in estrogen receptor-negative breast tumors as well as HER2-positive tumors. Such expression is correlated with a low survival rate in breast cancer patients, which might be ascribed to the essential role of TRPV6 in driving abnormal tumor cell proliferation. Importantly, tamoxifen, the widely used drug in breast cancer therapy, is more sensitive in reducing cell viability when TRPV6 is silenced (58). Interestingly, tamoxifen also functions as a negative modulator of TRPV6 as it reduces the calcium influx mediated by TRPV6 (59). Therefore, TRPV6 could be a potential drug target that alleviate chemotherapeutic resistance in breast cancers. Though TRPV1, TRPV4, and TRPV6 all mediate calcium fluxes in breast cancer cells, their unique and even opposite functions in breast cancer progression suggest a complicated network of calcium signaling in modulation of tumor cell functions, which requires further efforts to elucidate. Understanding the specific mechanism of each TRPVs would benefit precise targeting strategies for treating breast cancers, especially in the condition with large amount of extracellular calcium pools, like the bone metastatic niche.

#### TRPVs in Osteoclasts

TRPVs have been well-studied in osteoclast differentiation and activation. Except TRPV3, TRPV1, TRPV2, TRPV4, TRPV5, and TRPV6 are all reported to modulate osteoclasts formation or functions. Multiple studies reported that TRPV1 is required for osteoclastogenesis and bone resorption. TRPV1 mediated calcium influx is accompanied with cannabinoid receptors (CB) activation in osteoclasts, including CB1 and CB2. Intriguingly, CB1 and TRPV1 facilitates osteoclast differentiation while CB2 inhibits osteoclast activation (45). Nevertheless, TRPV1 deficient mice exhibit largely reduced osteoclast numbers and increase bone mass *in vivo*. Further analysis reveal a reduction of intracellular calcium levels and calcium oscillations in osteoclast precursors stimulated with RANKL (46). As calcium oscillations and the following activated NFATc1 are the major signals for osteoclastogenesis (24), TRPV1 seems to act as one of the early-stage determinants for osteoclast differentiation. TRPV2 is another TRPV that benefits calcium oscillations during osteoclast differentiation. The expression of TRPV2 is gradually increased during osteoclastogenesis (47), suggesting that TRPV2 is an essential calcium channel to sustain the early-stage calcium signals in osteoclasts. Importantly, the expression of TRPV2 is even enhanced in bone marrow biopsy specimens from patients suffering multiple myeloma (MM) compared to healthy controls, which is correlated with a poor clinical outcome of MM patients accompanied with enhanced osteoclast activation (48). Unlike TRPV1 and TRPV2, TRPV4 and TRPV5 are reported as critical modulators of osteoclast differentiation and activation in the late stage. TRPV4 deficient mice also exhibit significantly reduced osteoclasts number and enhanced bone mass. Interestingly, though osteoclast differentiation is restricted in TRPV4 deficient cells, this defect is more prominent in large or late-stage differentiated osteoclasts (52), suggesting TRPV4 mainly functions at the late-stage of osteoclastogenesis. Further analysis indicates that together with calcium oscillations in the early stage, TRPV4 induced calcium influx sustain NFATc1 activation and therefore maintain osteoclast differentiation. The same author further identified a gain of function mutant (R616Q/V620I) of TRPV4 and found the mutated mice show opposite phenotype compared to TRPV4 deficient mice (53), further confirming the importance of TRPV4 in modulation of osteoclast activation. Intriguingly, nearly 70 TRPV4 mutants were identified and cause disease in human patients (54), from which most of them causing skeleton dysplasia are gain of function mutants, confirming the significance of TRPV4 in modulation of osteoclastogenesis in mice. Opposite to TRPV4, TRPV5 deficiency leads to an increase of osteoclast size and number, however, the bone resorption are totally blocked in TRPV5 deficient osteoclasts (55). As TRPV5 is mainly localized at the ruffled border membrane in osteoclasts (56), it is reasonable that TRPV5 is essential for the function of mature osteoclasts. The enlarged osteoclast size could be due to compensation for bone resorption, similar as cathepsin K deficiency (105). Interestingly, human cells knocking down TRPV5 after osteoclast maturation leads to an enhanced bone resorption, opposite to the phenotype observed in TRPV5 knockout osteoclasts in mice (56). This could be due to the different stage they silenced TRPV5 or species variations. Nevertheless, TRPV5 is an essential modulator of osteoclast activation in the late stage. TRPV5 and TRPV6 are highly homologous in TRPV subfamily, with 75% homology in amino acids. TRPV6 deficiency also leads to a large osteoclast size and an increase in osteoclast numbers. Unlike TRPV5, TRPV6 knockout osteoclasts show a largely increase bone resorption (60), suggesting TRPV6 is clearly a negative modulator of osteoclast differentiation and activation. TRPV5 and TRPV6 share high similarity and distinct from other TRPVs, however, exhibit different functions in osteoclasts. As TRPV6 does not whereas TRPV5 does affect calcium oscillations during osteoclast differentiation, the mechanism of these two channels in osteoclasts should exhibit their unique features that has not been described in other systems, which required further studies to clarify.

Taken together, TRPVs play essential roles in osteoclast differentiation and activation in a stage-dependent manner. Considering the expression of TRPVs is dynamically modulated during osteoclast differentiation or in pathological conditions, it will be interesting to first analyze the expression profile of TRPVs in osteoclasts as well as tumor cells during bone metastasis. This might give the evidence how these TRPVs differently modulate either tumor progression or osteoclast activation, two essential aspects for bone metastasis. Also, unlike TRPCs, most TRPVs reported show opposite functions between tumor cells and osteoclasts, which TRPVs restrict tumor progression and are required for osteoclast activation. Interestingly, TRPV4 itself could both promote tumor cell death and metastasis, indicating that TRPVs might play totally different roles in certain scenario, and raising the possibility that certain TRPV, like TRPV4, would benefit both tumor cell invasion and osteoclastogenesis in the context of bone metastasis, which requires further efforts to elucidate.

### TRPMs

#### TRPMs in Breast Cancers

The mammalian TRPM subfamily contains eight members, from TRPM1 to TRPM8. TRPMs have also been recognized as important modulators in multiple cancers progression (106), from which TRPM2, TRPM7, and TRPM8 have been shown to promote breast cancer development. TRPM2 has been well-recognized to promote cell death and tissue injury (107–109), however, improve the cell viability in breast cancer cell lines (61). Silencing TRPM2 in MCF-7 and MDA-MB-231 breast cancer cell lines induces significantly DNA damage compared to that in MCF-10A, the widely used non-cancerous breast cells. Interestingly, calcium influx is not significantly affected when TRPM2 is inhibited in breast cancer cells (61), indicating TRPM2 would not regulate bone metastasis in a calcium microenvironment dependent manner. TRPM7 is well-studied and modulates numerous functions in breast cancer progression, especially in cancer metastasis (62–64, 110). Two SNPs of TRPM7 have been shown to associate with breast cancer in Han population of northeast China (111), indicating the importance of TRPM7 in breast cancers progression. Indeed, Kaplan–Meier analysis in breast cancer patients found that the high expression of TRPM7 is significantly correlated with recurrence-free survival and distant metastasis-free survival in breast cancers (64). Further analysis show that silencing TRPM7 reduces breast cancer cells migration and metastasis by regulation of myosin II–based cellular tension and thus cell movement (112). Mechanistically, the kinase domain of TRPM7 is mainly responsible for modulation of breast cancer cells migration. Importantly, TRPM7 mediated calcium signals further modulate EMT in breast cancer cells, which TRPM7 deficiency specifically reduces EGF-induced STAT3 phosphorylation and the expression of Vimentin, suggesting that TRPM7 is required for maintaining a mesenchymal feature in breast cancer cells (110). Except TRPM7, TRPM8 has also been reported to modulate EMT in breast cancer cells. Overexpression of TRPM8 remarkably increases MCF-7 migration and the shape change in 3D sphere formation. Whereas, silencing TRPM8 largely reduces migration and the shape switch in MDA-MB-231 cells (66). Taken together, TRPMs most likely regulate breast cancer metastasis, though one study also indicate TRPM7 regulates breast cancer cell proliferation (65). And, it will be reasonable to further analyze how the modulation of metastasis by TRPMs eventually regulates bone metastasis.

#### TRPMs in Osteoclasts

Unfortunately, so far little has been shown of TRPMs in modulation of osteoclast differentiation and activation. Considering TRPMs could be regulated by environmental changes of ATP, PH, heat, lipids, and also associate with other calcium channels to modulate calcium homeostasis (96), it is highly possible that TRPMs play essential roles in osteoclast differentiation and activation. Understanding the functions and mechanism of TRPMs both in breast cancer cells and osteoclasts will be beneficial for clarifying the importance of calcium microenvironment acting as a vicious cycle for bone metastasis, which would provide new potential and efficient drug targets for treating breast cancers.

### TRPA1, TRPPs, and TRPMLs

Compared to TRPC, TRPV, and TRPM subfamilies, little has been known about the other mammalian TRPs in modulation of breast cancers progression and osteoclasts activation, including TRPA, TRPP, and TRPML subfamilies. The mammalian TRPA subfamily only contains one member, named as TRPA1. The mammalian TRPP subfamily contains TRPP2, TRPP3, and TRPP5. And the mammalian TRPML subfamily contains TRPML1, TRPML2, and TRPML3 (96).

TRPA1 has been shown to be most highly upregulated among all of the TRPs in invasive ductal breast carcinoma, indicating TRPA1 promotes breast cancer progression. Indeed, TRPA1 accelerates breast cancer development in two routes. TRPA1 both enhances tumor growth and reduces chemo-sensitivity through mediating calcium influx dependent anti-apoptotic pathways (67). Similarly, TRPP2 has also been shown to promote drug resistance in breast cancers. Silencing TRPP2 does not affect the cell viability of breast cancer cells but impressively promotes the sensitivity of paclitaxel in treating MDA-MB-231 cells. TRPP2 is highly phosphorylated in breast cancer cells and treatment with paclitaxel further increases the phosphorylation level, which could be one of the mechanisms for the chemo-resistance (68). All in all, considering the drug resistance by TRPA1 and TRPP2 reported in breast cancers, these two channels probably are essential for tumor development in the late-stage, like bone metastasis. Their exact functions and mechanism in bone metastasis, especially in tumor induced osteoclastogenesis and osteolysis require further studies to elucidate.

TRPML1 is the only known cation channel in the TRPML subfamily (113). It has been shown that TRPML1 modulates calcium influx in lysosomes and is essential for osteoclastogenesis and bone resorption (69). Trpml1 deficient mice exhibit largely reduced osteoclasts and enhanced bone mass. *In vitro* analysis revealed that Trpml1 is required for lysosomal functions and therefore osteoclasts activation, probably via modulation of lysosomal calcium signals, one of the important sources for calcium oscillations and NFATc1 activation in osteoclasts. TRPML1 mediated lysosomal functions are also important in breast cancer development (70). TRPML1 is highly expressed in the triple negative breast cancer cells. Knocking down TRPML1 prevents lysosomal ATP exocytosis and therefore magnificently reduces tumor growth and migration. However, it is not clear whether TRPML1 promotes breast cancer progression in response to calcium signals. Considering the calcium mediated lysosomal functions and the related change of cellular metabolism mediated by organelle contacts are recently one of the most impressive fields in cancer development as well as multiple physiological and pathological functions (114), it would be very interesting to understand the potential promotion of bone metastasis by TRPML1-mediated lysosomal calcium cascades in breast cancer cells and osteoclasts.

## Voltage-Gated Calcium Channels (VGCCs)

The voltage-gated calcium channels are mostly permeable for calcium influx, with an extremely slight permeable for sodium ions in physiological conditions. VGCCs are mostly studied in excitable cells (115), like neurons and muscles, however, have also been shown to play essential roles in non-excitable cells (116), including breast cancers and osteoclasts. The activation of VGCCs requires membrane depolarization and mediates calcium influx to transduce downstream signals (117). VGCCs contain ten members, including Ca_v_1.1, Ca_v_1.2, Ca_v_1.3, Ca_v_1.4, Ca_v_2.1, Ca_v_2.2, Ca_v_2.3, Ca_v_3.1, Ca_v_3.2, and Ca_v_3.3. Depending on the cell types, VGCCs mediated calcium influx activates a variety of downstream targets for modulation of cellular functions.

The importance of VGCCs in modulation of breast cancers progression has been revealed by using an engineered VGCC lacking inactivation (Cec) (118), which triggers massive calcium influx and cell death in breast cancer cells but not in MCF-10A, the non-tumor human mammary epithelial cells. Importantly, the primary breast tumors generated by MDA-MB-231 are significantly shrank 2 weeks after infected with lentivirus containing Cec, indicating that activating VGCCs will be beneficial for the treatment of breast cancers. However, three VGCCs reported are all believed to promote cell proliferation or tumor growth in breast cancers (71, 73, 74, 119), including Ca_v_1.3, Ca_v_3.2, and Ca_v_3.3. Whereas, Ca_v_3.1 has been shown to act as a tumor suppressor gene in breast cancer cells by retarding proliferation and enhancing apoptosis (72), yet its exact role in tumor growth has not been investigated. Note the efficiency of VGCCs in promotion of breast cancers requires the constant activation of the channel, like the stimulation by extracellular pressure. For instance, cells only expressing Cec, the engineered Ca_v_1.2 lacking inactivation, but not Ca_v_1.2, largely induces cell death in breast cancers (118). Therefore, further studies are required to identify the stimuli for constant activation of VGCCs in breast cancers, or other tumor cells during bone metastasis, which is the important precondition for regulation of calcium homeostasis by VGCCs acting as a vicious cycle in bone metastasis.

## Store-Operated Calcium Entry (SOCE)

SOCE is an ubiquitous mechanism in non-excitable cells to modulate calcium homeostasis with important biological functions (120). During the last decade, the most important advance in SOCE field is the identification of ER-resident STIM1 (121–123) and plasma membrane (PM)-located ORAI1 (124–126) as two major components for SOCE activation. Their homology, STIM2, ORAI2, and ORAI3, have also been shown to participate in SOCE, yet the functions are minor or with controversy compared to STIM1 and ORAI1 (127). SOCE activation is a multistep process that requires the conformational change of STIM1 and ORAI1 (28). Particularly, STIM1 is inactivated in the ER by association with calcium via the EF-hand domain located in its N-terminal region. The decline or depletion of calcium in the ER due to either promotion of calcium release or reduction of calcium reuptake therefore leads to the conformational change and thus oligomerization of STIM1. The oligomerized STIM1 then redistributes to the ER-PM contact with the help of the cytoskeleton system, where it associates with the clustered ORAI1 and forms the channel for extracellular calcium entry, which ultimately refills the ER calcium storage to sustain calcium signals in the cells. SOCE therefore is an essential and specific process to maintain calcium homeostasis in cells when or after cells were activated by extracellular stimuli, which is essential for biological functions. Indeed, Stim1 or Orai1 deficiency in mice is embryonic lethal and numerous STIM1 or ORAI1 mutants have been identified in humans exhibiting disorders of calcium influx in cells. Loss- and gain- of function mutants both result in multiple severe disease in human patients (128), including immune disorders and skeleton abnormal development.

### SOCE in Breast Cancers

Recent evidence further reveals the importance of SOCE in modulation of cancer progression, including breast cancers (75). SOCE is closely related to breast cancer metastasis, especially bone metastasis. Several studies found that SOCE facilitates migration and metastasis in breast cancers mainly via three routes. Yang et al. show that knocking down either STIM1 or ORAI1 in MDA-MB-231 decreases the invasion while overexpression of STIM1 and ORAI1 together in MCF-10A enhances it (76). This could be due to the impairment of assembly and disassembly of focal adhesions in STIM1 or ORAI1 deficient cells. Importantly, pharmacological inhibition of SOCE by SKF96365 significantly reduces breast cancer cell metastasis, giving an evidence that targeting SOCE could be a potential strategy for treating tumor metastasis. SOCE is also essential for the enolase-1 (ENO-1) exteriorization to the cell surface (77). The exteriorized ENO-1 modulates pericellular proteolysis and thus allows cells to invade tissues (129). Therefore, SOCE can also promote the migration and invasion of breast cancer cells via mediating the translocation of ENO-1 to cell surfaces. Finally, SOCE promotes TGFβ-induced EMT during breast cancer progression (78). Both STIM1 deficiency and pharmacological inhibition of SOCE by YM58433 reduce the expression of Vimentin but enhances the expression of E-cadherin stimulated with TGF-β in breast cancer cells, indicating that SOCE is required for maintaining the epithelial conditions in breast cancer cells and thus modulates tumor cell migration. Interestingly, one study found that SOCE is also slightly required for TGF-β induced cell cycle arrest in breast cancer cells (79). Dependent on the tumor stages, TGF-β signals have been shown to have opposite functions in breast cancer cells (130). Therefore, SOCE might also has similar effects that SOCE and TGF-β signals synergistically restrict breast cancer cell proliferation in the early stage, whereas in the late stage, SOCE modulates TGF-β induced EMT and tumor metastasis. The importance of SOCE in modulation of breast cancers progression has also been revealed by analyzing the clinical relevance in human samples. Both STIM1 and ORA11 express highly in breast cancer cells and their high expression are correlated with tumor aggressiveness and poor prognosis of breast cancers (131). In addition, three studies reported multiple SNPs of ORAI1 in breast cancers, which were predicted to associate with tumor malignancy (132–134). Overall, SOCE activation has been shown to promote breast cancer progression, especially via enhancing the tumor metastasis.

### SOCE in Osteoclasts

SOCE has also been shown to be a critical signal for calcium oscillations during osteoclast differentiation. Knocking down Stim1 in pre-osteoclasts dramatically reduces calcium oscillations (80), the essential signals for osteoclast differentiation. Orai1 deficiency also shows reduction of SOCE, impairment of NFATc1 translocation and defect of pre-osteoclasts fusion as well as osteoclastogenesis (81–83).

Combined with the observation of SOCE in modulation of cancer metastasis and osteoclastogenesis, it will be reasonable to speculate that SOCE activation would lead to bone metastasis. Indeed, one study show that the SK3, a potassium channel, associates with ORAI1 in lipid rafts and controls the constitutive calcium entry and thus bone metastasis in breast cancers (84). Another study found that SGK1 is essential for ORAI1 expression and therefore modulates calcium entry and osteoclastogenesis, which ultimately benefits bone metastasis (135). Therefore, SOCE could be essential signals for bone metastasis and targeting SOCE would be a potential strategy to treat this disease. However, since SOCE activation is widely required in non-excitable cells, targeting SOCE itself would lead to severe side effects. The good news is that multiple modulators have been identified to either promote or restrict SOCE activation (136–144), which would be potential targets for treating breast cancers without totally blocking SOCE activation. Further studies are required to clarify the exact roles and mechanism of these SOCE modulators in breast cancers progression, especially the bone metastasis.

### P2X Receptors

P2X receptors are ligand-gated ion channels that are principally activated by ATP. So far seven members of this subfamily have been identified, which are numbered P2X1 through P2X7. Activation of P2X receptors by ATP would lead to trimerization of these receptors for cations entry, such as sodium or calcium ions. Both homo-trimers and hetero-trimers of these receptors have been reported (145). Similar as VGCCs, P2X receptors modulate calcium entry mostly in excitable cells, however, have also been shown to participate in regulation of tumor progression and osteoclastogenesis, especially the P2X7 receptor.

### P2X7 in Breast Cancers

The P2X7 receptor modulates proliferation, apoptosis, migration, invasion and metastasis in breast cancers. The P2X7 receptor could be activated by ATP that is rich in the tumor microenvironment, leading to the downregulation of E-cadherin and upregulation of MMP-13 mediated by the PI3K-AKT cascade in T47D breast cancer cells (85). This is one of the mechanisms shown to promote breast cancer cell invasion by the P2X7 receptor. Activation of the P2X7 receptor also changes the morphology of MDA-MB-435 cells, which prolongs the cell shape facilitating cell migration. Interestingly, P2X7 mediated cell migration but not cell extension is largely reduced in SK3 deficient cells. Nevertheless, P2X7 enhanced cell invasion could also be mediated by the SK3 channel (86). A recent study further suggested that P2X7 promotes cell migration and metastasis via increasing the extracellular vesicles production in tamoxifen-resistant breast cancers (87), further indicating the possibility to target P2X7 as a strategy for alleviating drug resistance in breast cancers. Except cell invasion, the P2X7 receptor also increases cell proliferation and reduces cell apoptosis in breast cancer cells. Knocking down P2X7 in MCF-7 cells almost blocks cell viability and significantly increases apoptosis (88). One study further found that P2X7 exists a distinct conformational form that restricts the large pore opening in tumor cells, named as non-pore functional P2X7 (nfP2X7). The nfP2X7 signal is essential for breast cancer cell survival and proliferation but has limited calcium entry therefore declines cell death (89). It is interesting to observe that nfP2X7 in the breast cancer cells has similar functions as P2X7 except calcium entry, which indicates that modulation of the calcium microenvironment would directly affects breast cancer cell viability as we proposed initially. It would be more interesting to analyze whether cancer cells have these type of non-functional P2Xs in cancer cells migrated to the osteolytic tissues where amounts of calcium ions exist, sustaining the benefits from calcium signaling for tumor growth but limiting the excessive calcium entry that leads to cancer cell death.

### P2X7 in Osteoclasts

In addition to breast cancer cells, the P2X7 receptor is also well-studied in modulation of osteoclastogenesis. The P2X7 receptor links the extracellular stimulus and osteoclast activation, which the mechanical and other stimuli leads to nucleotides release, including ATP, and activates osteoclastogenesis via ATP mediated activation of P2X7 and the downstream NF-κB cascade (90). Interestingly, osteoclasts are normally fused and differentiated in P2X7 knockout mice (91), indicating that P2X7 is maintained inactive in physiological conditions but largely activated in pathological conditions. Indeed, it has been reported that compared to WT mice, the P2X7 deficient mice exhibit largely reduced bone mass and increased osteoclast numbers in OVX-mediated osteoporosis model, but not in the SHAM control (92). Another study further revealed that P2X7 drives pre-osteoclast fusion in response to amount of ATP stimulation (93), indicating that P2X7 might be a critical signal for pathological osteoclast activation and bone remodeling as damaged bone would release a large number of ATP and other nucleotides. In humans, several SNPs of P2X7 have been identified to associate with osteoporosis in postmenopausal woman (146, 147) as well as the risk of fracture (147, 148). Pharmacological inhibition of P2X7 significantly inhibits human osteoclasts formation (94), suggesting the importance of P2X7 activation in modulation of bone disease in human.

Taken together, P2X7 plays important roles in osteoclast activation specifically in pathological conditions, raising the possibility that P2X7 would play essential roles in bone metastasis. Actually Zhou et al. have shown adenosine nucleotides promotes breast cancer growth and bone metastasis in a high dosage (95). Interestingly, this study found that ATP mediated activation of P2X7 inhibits MDA-MB-231 cells migration, raising a possibility that P2X7 might be essential for tumor cell residence in osteolytic niche after bone metastasis, which requires further studies to investigate.

## Extracellular Calcium Entry in Osteoblasts

Calcium homeostasis are also important signals in modulation of osteoblast differentiation and functions. Till now, though not as much as those reported in breast cancer cells and osteoclasts, multiple calcium channels have also been reported to modulate osteoblast proliferation, differentiation, migration and mineralization, including TRPV1, TRPV4, TRPM7, TRPP2, Cav1.2, ORAI1/SOCE, P2X1, and P2X7. He et al., reported that TRPV1 deficient BMSCs exhibited impaired osteoblast differentiation and mineralization *in vitro*. As a result, TRPV1 deficiency leads to delayed fracture healing in the pathological mice model (46). TRPV4 has also been indicated in regulation of osteoblast activity, which TRPV4 is highly induced in differentiated osteoblasts and essential for calcium oscillations in osteoblasts (149). Though the importance of TRPV4-induced calcium oscillations in regulation of osteoblast activity have not been well-clarified yet, considering calcium oscillations are one of the important features in mature osteoblast and osteocytes in response to mechanical force (150), it would be reasonable to speculate that TRPV4 is essential for mature osteoblast activity, which requires further efforts to elucidated. Similar as TRPV4, TRPM7 is also upregulated during osteoblast differentiation. TRPM7 deficiency has been reported to result in defects in osteoblast proliferation, differentiation and mineralization, however, such functions might be via not only calcium but also magnesium entry (151, 152). TRPP2 is another TRP that has been reported to be essential for osteoblast differentiation and mineralization. The TRPP2 conditional knockout mice exhibit significantly reduced bone mass both in trabecular and cortical bone (153). These observations raise the importance of TRPs in modulation of osteoblast activity as all of the TRPs reported are require for osteoblast differentiation and mineralization. In addition to TRPs, Cav1.2 (154), ORAI1 (155, 156), and P2X1 (157) have also been shown to promote osteoblast differentiation and mineralization *in vitro* and *in vivo*, suggesting extracellular calcium entry is require for the maintenance of osteoblast activity.

While abnormal osteoblastogenesis is more frequently observed in prostate cancers driven bone metastasis, the major function of osteoblasts during bone metastasis in breast cancers has been believed to enhance RANKL expression but reduce OPG expression stimulated by tumor cells, and therefore facilitate osteoclastogenesis and tumor cells metastasis (7). Though extracellular calcium entry is required for osteoblast differentiation and maturation, it would be more important to explore whether calcium channels modulate RANKL/OPG expression in osteoblasts. Interestingly, high dietary calcium administration in mice leads to enhanced osteoblastic bone formation and slightly but significantly reduces RANKL/OPG ratio in bone extracts (158). Another study further finds that TRPV1 activation promotes OPG expression but not affects RANKL expression, which leads to a reduced RANKL/OPG ratio (159), similar as the dietary calcium administration. Also, the gain of function mutated Cav1.2 mice (Prx1-Cre driven) exhibit increased serum OPG concentrations and the isolated BMSCs show reduced RANKL/OPG expression. Importantly, the Cav1.2 mutated calvaria osteoblasts exhibit defects in promotion of osteoclastogenesis in the co-culture system *in vitro* (154). Taken together, it seems that extracellular calcium entry in osteoblasts would suppress RANKL/OPG ratio, osteoclastogenesis and therefore benefit bone formation. It would be reasonable to assume that extracellular calcium entry would act as a negative factor in the scenario of osteoblasts-mediated bone metastatic niche formation in breast cancers. However, as the reduced RANKL/OPG ratio reported is slightly modulated by extracellular calcium, further studies are required to clarify whether the extracellular calcium modulated RANKL/OPG ratio produced in osteoblasts would be compensated by other major signals like cytokines secreted from breast cancer cells during bone metastasis, and therefore plays minor effects in the whole vicious cycle.

## Calcium Channels and Bone Metastasis in the Specific Subtype of Breast Cancers

One of the largest issues in tumors is the heterogeneity that tumors are a mixture of different type of cells with different molecular makers (160). Thanks to the efforts made by numerous researchers, several classifications have been developed to categorize breast cancers (161, 162), including the immunohistochemical subtype characterized by the expression of estrogen receptors (ER), progesterone (PR) and epidermal growth factor receptor 2 (HER2). According to the expression of these three receptors, the breast cancers can be categorized as Luminal A (ER+PR+HER2-), Luminal B (ER+PR+HER2+), HER2+ (ER-PR-HER2+), and triple negative breast cancers (TNBC, ER-PR-HER2-). These four subtypes of breast cancers also exhibit different potency of bone metastasis (160). Overall, the luminal and Her2+ subtype of breast cancers exhibit high potency of bone metastasis compared to the basal-like tumors, the major components (around 75%) in TNBC subtype. Importantly, no luminal subtype has been observed in TNBC subtype. In another word, ER/PR+ and Her2+ tumors show high potency of bone metastasis compared to the TNBC subtype.

Interestingly, the expression of calcium channels is dynamically regulated in these three subtypes and have been well-summarized recently (163, 164). Particularly, TRPC1, STIM1, and ORAI1 are downregulated in luminal and Her2+ subtypes but upregulated in the TNBC subtype. Whereas, the expression of Cav3.2 is upregulated in luminal and Her2+ subtypes but downregulated in the TNBC subtype. These observations indicate that VGCCs might be essential signals in modulation of calcium homeostasis and therefore bone metastasis in luminal and Her2+ subtypes. In addition, one cannot exclude the possibility that the low expression/activation of TRPs and SOCE in luminal and Her2+ subtypes is the result of a negative feedback, which the over-activation of TRPs and SOCE strongly enhance calcium influx in breast cancers and thus bone metastasis, however, too much calcium burden in turn declines the expression of calcium channels (TRPs, STIM1, and ORA1) to balance the intracellular calcium homeostasis, which has been reported in other cation channels (165). In conclusion, further studies are required to utilize *in vivo* mice model or clinical samples but not breast cancer cell lines alone to elucidate the importance of VGCCs/TRPs/SOCE in luminal and Her2+ subtypes in the context of bone metastasis.

All in all, all of the four calcium channels have shown their potency in modulation of breast cancers progression, including tumor cells viability and migration, osteoclasts activation, and bone metastasis. Further studies are required to elucidate that (1) how does these channels respond to the calcium microenvironment and tumor progression during bone metastasis? (2) Do these channels synergistically or independently modulate bone metastasis? (3) Do these channels modulate bone metastasis in a time- and space- dependent manner? (4) Do these channels dynamically modulated in specific subtypes of breast cancers and related bone metastasis? It is worth noting that since tumor cells and osteoclasts are non-excitable cells, SOCE would be an extremely important modulator for bone metastasis as SOCE mainly regulates calcium homeostasis in non-excitable cells. Moreover, SOCE normally modulates biological functions synergistically with cascades declining or depleting the ER calcium storage, therefore modulation of SOCE activity would not heavily hurt physiological activities of cytosolic calcium signals in a short range, leading to limited side effects in treating bone metastasis.

## Treating Bone Metastasis By Considering Systemic Calcium Homeostasis

The calcium channels have already been shown to be critical modulators for breast cancers progression and osteoclasts activation. Multiple reports have shown the potency of agonists or antagonists targeting calcium transporters in treating cancers both in mice models and in pre-clinical studies (15, 166). Also, studies summarized above mentioned that several agonists or antagonists targeting these channels would affect breast cancers progression in *vitro* and *in vivo*, raising the possibility that these compounds could be potential drugs for treating breast cancers and even bone metastasis. Here we will not overview again these agonists or antagonists, but focus more on the known drugs treating bone metastasis in sight of modulation of systemic calcium homeostasis, including bisphosphonates, and denosumab.

Bisphosphonates are a family of drugs that suppress osteoclasts-mediated bone resorption and alleviate abnormal bone loss in multiple diseases (167). Now it has been well-recognized that bisphosphonates inhibit bone resorption mainly via four routes, including preventing the recruitment, inhibiting the adhesion, shortening the lifespan and reducing the activity of osteoclasts (168). Bisphosphonates were discovered for mainly treating osteoporosis but have also been shown to be effective in osteoclasts-related disease, including bone metastasis in breast cancers. Bisphosphonates exhibit dual functions in bone metastasis. On the one hand, it alleviates bone loss and relevant bone pain therefore suppresses serum calcium concentrations. On the other hand, bisphosphonates have been reported to induce apoptosis and suppress invasion in tumor cells therefore ameliorate tumor growth (169). Importantly, treatment of bisphosphonates in patients not only alleviates bone loss but also results in hypocalcemia, a status due to reduced calcium loss from bones (167). So far the reduction of serum calcium concentrations due to bisphosphonates treatment have been well-recognized. The secondary effects of this reduction that the decline of extracellular calcium microenvironment would counteract on the activity of tumor cells and osteoclasts require further studies to elucidate. This is important as hypocalcemia is a short-lasting event and serum calcium concentrations would be recovered due to a negative feedback that parathyroid hormone is increased for upregulation of calcium absorption (170). Considering the serum calcium concentration would play crucial roles in bone metastasis via the calcium channels, the duration for administration of calcium or Vitamin D to treat hypocalcemia due to bisphosphonates treatment might need to be reconsidered in patients suffering bone metastasis.

Denosumab is another drug treating bone loss by targeting osteoclasts-mediated bone resorption. Denosumab is an antibody targeting human but not rodent RANKL, the well-known essential ligands for osteoclastogenesis, and therefore is utilized to treat multiple osteoclasts-related disease, including bone metastasis (171, 172). Indeed, the scope of application of denosumab in clinic trial includes osteoporosis and multiple types of cancers developing bone metastasis. Similar as bisphosphonates, administration of denosumab efficiently alleviates bone loss and reduces bone metastasis. In a pre-specified interim analysis of giant cell tumors, 99% patients have been shown to respond to exhibit no disease progression after 12-months treatment (173), suggesting the high efficiency of denosumab in treating osteoclasts-related tumor progression. Interestingly, administration of denosumab also leads to hypocalcemia, but with a pretty low incidence in osteoporosis while a relatively high percentage in cancers (172). This might be due to the extremely high activation of osteoclasts and hypercalcemia in cancer patients. Further studies are required to clarify if tumors are more coordinated to hypercalcemia that leads to its malignancy including bone metastasis, and treatment of denosumab could nicely and specifically block the vicious cycle raised by the abnormal calcium homeostasis in bone metastasis.

Other inhibitors targeting bone resorption or RANKL-RANK signaling have also been reported to be effective in treating tumor progression and bone metastasis, including OPG-Fc (172), RANK-Fc (172), calcitonin (174), etc. Based on the observation of bisphosphonates and denosumab in treating bone metastasis, it will be reasonable to speculate that all of the other inhibitors modulation of hypercalcemia in cancer patients would likely lead to hypocalcemia. Therefore, it would be interesting to clarify if these inhibitors also alleviate tumor progression by affecting the calcium microenvironment during bone metastasis, which might provide efficient strategies of combinational therapies to synergistically treat cancers.

## Conclusion and Perspective

Hypercalcemia has been recognized as the results of bone resorption during tumor progression. Raising of serum calcium concentrations in patients suffering hypercalcemia would lead to multiple disorders, which severely affects human health and even leads to mortality (19, 175). The importance of hypercalcemia in direct modulation of tumor progression has not been well-evaluated yet. Considering that the extracellular calcium and the related calcium channels have multiple functions in regulation of tumor progression and osteoclastogenesis, it would be reasonable to hypothesize that hypercalcemia in cancer patients further aggravates the tumor progression via the abnormal calcium homeostasis forming a vicious cycle among tumor cells and osteoclasts during bone metastasis, which could be one of the reasons to explain the high malignancy of tumor progression in patients suffering hypercalcemia. TRPs, VGCCs, SOCE and P2Xs are four major channels for calcium entry and play important roles in tumor cells proliferation, survival, migration and metastasis. Also, these four channels modulate osteoclast differentiation and activation in certain scenarios. Though different functions of these four channels have been observed in modulation of the activity in cancer cells and osteoclasts, most of them show their capacity in promoting tumor progression and osteoclast activation. Therefore, further efforts are required to elucidate the exact functions and mechanism of these four channels in bone metastasis, especially SOCE due to its specificity for calcium entry and its omnipresence in non-excitable cells. Understanding the vicious cycle of calcium homeostasis in bone metastasis mediated by these calcium channels would further provide potential combinational strategies together with the known chemotherapeutic treatments to treat cancers, including targeting the functional calcium channels or modulating the serum calcium concentrations.

## Author Contributions

ZYa and ZYu generated the concept and wrote the manuscript. XM and ZX searched the literatures and reviewed the manuscript. All of the authors approved the final manuscript.

### Conflict of Interest

The authors declare that the research was conducted in the absence of any commercial or financial relationships that could be construed as a potential conflict of interest.
